# Fok-I, Bsm-I, and Taq-I Variants of Vitamin D Receptor Polymorphism in the Development of Autism Spectrum Disorder: A Literature Review

**DOI:** 10.7759/cureus.3228

**Published:** 2018-08-29

**Authors:** Sharmi Biswas, Bushra Kanwal, Charan Jeet, Robert S Seminara

**Affiliations:** 1 Pediatric, California Institute of Behavioral Neurosciences & Psychology, Fairfield, California , USA; 2 Lombardi Comprehensive Cancer Center, Georgetown University Medical Center, Washington DC, USA; 3 Department of Research, California Institute of Behavioral Neurosciences & Psychology, Fairfield, USA; 4 Neuroscience, California Institute of Behavioral Neurosciences & Psychology, Fairfield, USA

**Keywords:** autism spectrum disorder, vitamin d, vitamin d receptor polymorphism, fok-i, bsm-i, taq-i, neurosteroid

## Abstract

The role of vitamin D in the development of autism spectrum disorder (ASD) is of intensified interest in medical science in recent years. Vitamin D has a significant role in neurogenesis, neuroprotection, and neurodevelopment. Due to the close association of vitamin D with the brain, it has been found that in the pathophysiology of several neuropsychiatric disorders vitamin D receptor (VDR) polymorphism plays a significant role. In this review article, we looked for a relation between VDR polymorphism and ASD. We systemically reviewed all the potential articles on the relation between VDR polymorphism and ASD. We found that several VDR variants FokI, BsmI, and TaqI polymorphisms are related to ASD. Even paternal VDR polymorphism can be a causative factor for ASD in the offspring. The relation between FokI (ff) genotype polymorphism and increased level of serum 1,25(OH)D3 in ASD patients is a very significant finding. Variation of ASD-related genotypes in different ethnic population raises a big question on whether the environmental factors also can do changes in human genotypes leading to ASD.

## Introduction and background

Autism spectrum disorder (ASD) defines a broad range of conditions portrayed by compromised social skills, repetitive behaviors, and deficits of speech and nonverbal communication. ASD affects >1% of children in the United States [1]. It is mainly diagnosed by the clinical signs and symptoms; in fact, the precise etiology continues to elude researchers [2]. Only 10%-35% of ASD patients have a major known risk factor, while the remaining cases appear to be more sporadic [3]. Since last decade, the prevalence of ASD has been increasing dramatically. Attention is now being given to possible environmental and genetic risk factors associated with ASD [4]. Based on the adolescent’s predominant symptom, there are different subgroups of ASD: autism, Asperger syndrome, and pervasive developmental disorder not otherwise specified. Individuals with ASD have difficulties in establishing relationships with others and expressing emotions while failing to conform to social expectations. Autism patients are more prone to psychiatric and metabolic dysfunction [5]. Further evidence even suggests autism may be of an inflammatory nature. Autoantibodies targeting the brain and glutathione subsets show that ASD may be associated with high levels of oxidative stress [6-8].

Since last decade, the prevalence of ASD has been increasing dramatically. Attention is now being given to possible environmental and genetic risk factors associated with ASD. Most commonly identified risk factors for ASD are a male gender, gene-environment reaction, children born in early spring, immigrant mothers, and paternal age [9-10]. Cannell, in 2008, revealed a hypothesis that low vitamin D level in fetal life and early childhood has a strong role in ASD [11]. Vitamin D exerts its effect not only at the cellular level, but it also affects the gene expression by inducing changes in vitamin D response elements. Autism-specific neural changes like overgrowth of the brain in neonatal life, retardation of brain growth in early childhood, and neurodegenerative changes in adults happen. In gestational hypovitaminosis, it also shows the overgrowth of the brain in the fetus which indicates a possible role of vitamin D in the pathophysiology of autism. In fact, animal studies show that vitamin D deficiency in fetal life has a drastic neuromodulatory effect on the offspring, causing structural and functional changes in the brain. Furthermore, the most recent breakthrough in vitamin D functionality has been neuroprotective-like effect exhibited during fetal development [9, 12-13].

## Review

Vitamin D in human body

**Figure 1:**
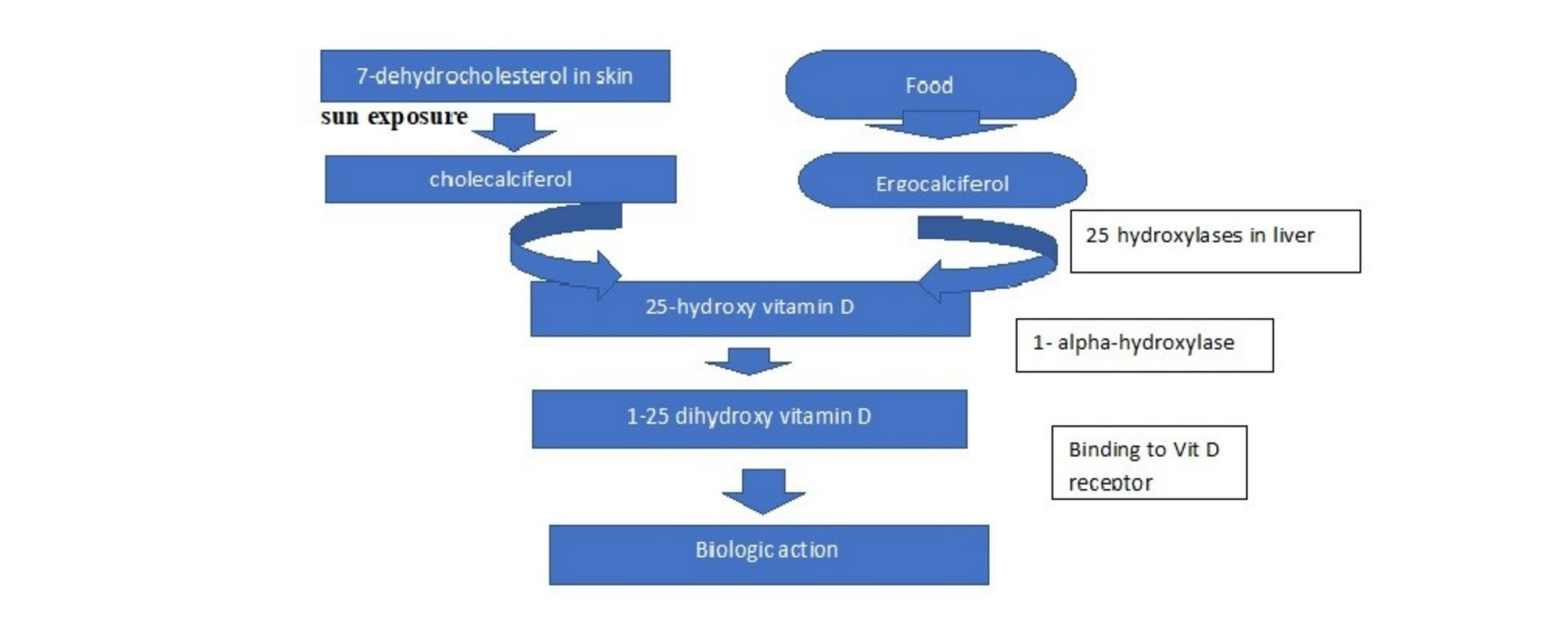
Vitamin D synthesis in human body.

Vitamin D is a prohormone though classified as a vitamin. In general, food is a rare source of vitamin D except for fish liver oil and plants like Solanum glaucophyllum. A significant source of vitamin D is through exposure of skin to ultraviolet B (UVB) radiation. On exposure to sun, 7-dihydroxy cholesterol of epidermis and dermis absorbs the UVB and gets converted to pre-vitamin D3. Through heat-induced isomerization, pre-vitamin D3 changes to vitamin D3 (Figure 1). Vitamin D3 is a biologically inert substance; it needs hydroxylation in the liver and kidney to change into its biologically active form 1,25-dihydroxy vitamin D3. Once formed, 1,25-dihydroxy vitamin D3 acts through a special nuclear receptor to conduct its biological functions including calcium and phosphate absorption in the intestine, mobilization of calcium in bone, and renal reabsorption of calcium. Vitamin D also plays an important role in other noncalcemic pathways of the body [14-15]. Vitamin D receptors (VDRs) were found in the primary target cells of enterocytes, osteoblasts, and distal renal tubular cells as well as in parathyroid gland cells, skin keratinocytes, promyelocytes, lymphocytes, colon cells, and in ovarian cells. Identification of VDR in these cells signifies the potential role of vitamin D there [14].

Genomic and nongenomic pathways of vitamin D receptor

Vitamin D follows two pathways of action—genomic and nongenomic. In the genomic pathway, Vitamin D binds with the VDR, a member of the steroid/thyroid superfamily of transcription factors. After binding to vitamin D, fetal VDR gets phosphorylated; then gets heterodimerized with the retinoid X receptor which binds to VDR elements (VDRE) within the genome to influence gene transcription [16-17]. A study done on 2,200 genomic positions of VDR confirmed that vitamin D is pleiotropic [18].

In the nongenomic pathway, vitamin D binds to the membrane-bound VDR, or a protein disulfide isomerase associated 3(PDIA3) protein. Following the signal transduction pathway, vitamin D causes the release of an intracellular influx of Ca2+ leading to the activation of protein kinase which eventually changes the phosphorylation of cellular proteins [16].

The study showed that the nongenomic pathway of vitamin D action has an abundant role in cellular proliferation and immune function [19]. In the mammalian brain including the human brain, both VDR and 1 alpha-hydroxylase were identified. VDR is present in the brain, spinal cord as well as in the areas which control motor function and behavior in animal models. VDR knockout mice showed significant changes in stereotypical behaviors like increased grooming activity which indicate the presence of VDR in the areas of the brain (hypothalamus, basal ganglia, limbic system) related to grooming activity [20-21].

Vitamin D functions

Vitamin D is the primary hormone for calcium and phosphorus regulation. It also plays a role in bone formation, resorption, mineralization as well as in the maintenance of neuromuscular function. Rickets and osteomalacia are two well-known conditions caused by vitamin D deficiency, but there are many other conditions reported related to vitamin D deficiency. Fifteen different types of cancer, osteoporosis, orthostatic hypotension, diabetes mellitus, hypertension, hyperlipidemia, inflammatory bowel disease, rheumatoid arthritis, multiple sclerosis are also reported being related with vitamin D deficiency [22].

Fetal brain and vitamin D

Vitamin D metabolite 25-hydroxyvitamin D (25-OHD) was first detected in the human cerebrospinal fluid (CSF) in 1980. A study showed that 25-OHD concentration is similar in human plasma and CSF which proves that vitamin D can easily cross the blood-brain barrier. Vitamin D dependent calcium binding protein (D-CaBP) increases calcium absorption in the intestine and D-CaBP also has been reported to be present in the human brain [23]. 1-Alpha-hydroxylase, the activating enzyme of vitamin D is found in the brain. Also, 25-hydroxylase and 24-hydroxylase (CYP24A1), which degrade the active biological form of vitamin D, are also present in the human brain [16]. Cytochrome P450 enzyme regulating vitamin D synthesis, CYP27B1 has also been detected in fetal and adult human brain. In the adult brain, CYP27B1 is present in neurons, glial cells with schizophrenia, expression in substantia nigra, supraoptic, and paraventricular nucleus of the hypothalamus. Distribution of CYP27B1 in adult brain proves that brain can synthesize the active metabolite 1,25(OH)2D3 [17, 24].

Vitamin D has a crucial role in pregnancy due to its primary role in developing brain of the fetus. Inadequate exposure to vitamin D causes several changes in the fetal brain which are responsible for adverse outcomes in brain function in later life. The significant role of vitamin D in fetal brain development has been widely accepted in the last decade and vitamin D is now being considered as a neurosteroid. Vitamin D influences the developing brain by its several endocrine functions via regulating extracellular calcium, inflammation-mediated cytokines, and glucocorticoids. By regulating these crucial factors in the fetal brain, vitamin D expresses its significant role in neurogenesis, neurodifferentiation, and neuroprotection since the initiation of a life [24]. A study was done on Bagg Albino (BALB/c) mice and showed that maternal vitamin D deficiency induced neuroanatomical alterations and revised gene expressions [25]. In fetal mice, vitamin D deficiency caused suppression of neural forkhead box protein P2(FoxP2) and tyrosine hydroxylase (TH), especially in females. Fox P2 is an essential gene for speech and language developments causing dyspraxia, difficulties in expressive and receptive language, as well as this is the pathway which gets hampered in some cases of ASD. FoxP2 knockout models showed delays in developments, altered motor function, and impaired cerebellar morphology [26-28].

There is a robust decrease in TH level in mice brain with reduced staining of TH proteins in substantia nigra which shows a strong relation between vitamin D deficiency and dopamine dysfunction. Studies showed that 1,25(OH)2D3 can increase TH and VDR expression in TH positive neurons of the substantia nigra in both human and rat fetal brain. Developmental vitamin D deficiency can change dopamine cell phenotype expression in fetal rat mesencephalon, dopamine-dependent locomotion, dopamine turnover, dopamine transporters, and several enzyme levels related to metabolism [29-32].

Vitamin D deficient rat fetuses also showed changes in brain structure like the increased size of the lateral ventricle, decreased crown-rump, and lambda- bregma length [25, 33]. There was an increased size of the lateral ventricle with other neuroanatomical changes for schizophrenia-like behaviors. In humans also schizophrenia is associated with increased lateral ventricular size [33-34].

Vitamin D deficiency in the prenatal and postnatal periods is related to some neuropsychiatric disorders in later life like schizophrenia, autism, and multiple sclerosis [16, 35]. Evidence showed that 25-OHD level <40 ng/ml in the gestational and early childhood period is linked with autism. Supplementation of vitamin D in pregnant mothers (5000 IU/day), infants, and children (1000 IU/day), significantly reduced the risk of incidence of autism from 20% to 5%. Association between high latitude and autism is prominent as it has been found that children who live in low UVB light, are at three times greater risk of having autism than the children from sunny areas. Babies born in late winter also showed increased rates of autism as the pregnant mothers spent significant time in low sunlight settings. Primary care providers should focus on maximizing vitamin D intake in pregnant and lactating women as well as in infants and young children; according to the Endocrine Society, the recommended level is up to 46 ng/ml. To prevent autism, pregnant and lactating women need to take 10,000 IU/day and a breastfeeding infant to 6 years old child should take 150 IU/lb/day to reach the 25-OHD level above 40 ng/ml [35].

Vitamin D receptor polymorphism in ASD

To execute functions, vitamin D needs to bind with VDRs present in the human body. The VDR receptor is present in the whole mammalian brain. The first expression of VDR happens in E12 in rat brain and on an embryonic day (E) 11.5 in mouse brain. The time of VDR expression coincides with the window period of brain development when there is reduced cell proliferation, and increased cell elimination occurs [35]. Studies found that VDR protein is present in multiple areas of rat and human brains such as the pontine-midbrain thalamus, hypothalamus, cerebellum, basal ganglia, amygdala hippocampus, olfactory system, and cerebral cortex (temporal, parietal, cingulate) [16, 17, 35]. Each VDR gene consists of nine exons and nine introns which can be located in the chromosome 12q13 [16, 36]. The VDR is a member of the neurosteroid family which signifies the role of VDR as a candidate gene for ASD [36-38]. Studies showed the association of several single nucleotide polymorphisms (SNPs) of VDR genes with ASD but the SNPs could be variable for different population [39]. Three haplotypes of VDR is associated with ASD which are GTTT, GTCT, ATCG. The haplotypes showed polymorphisms in four restriction enzymes like BsmI(G>A), FokI (C>T), ApaI(T>G), and TaqI(T>C). In ASD the more common haplotypes found was GTTT while ATCG and GTCT were found of lower frequencies in ASD patients [37]. Animal studies done on mice with knocked out VDR gene showed impairment of their behavior [35-37] as well as in hearing [38-39]. The most common promoter region of VDR which is related to multiple diseases is rs15568820 and rs4516035. But Coşkun et al. showed that autism disorder has no association with rs15568820. Restriction fragment length polymorphism (RFLP) assay done using different enzymes showed important polymorphisms in the VDR gene. In intron 8, there was detection of polymorphisms by the restriction enzyme Apa-I A/a(adenine>cytosine)(rs797532) and BsmIB/b(adenine>guanine) (rs1544410) [37, 40]. Several other polymorphisms also got detected which also affect gene expressions such as rs 731236(Taq 1) at exon 9 and rs11568829 Caudal type Homeobox 2(Cdx2) in the promoter region. Taq1 polymorphism can alter protein structure and binding specificity of VDR. The Cdx 2 polymorphism affects the transcriptional activity. The rs 2228570(Fok1) is in exon two which can alter the transcription initiation site due to polymorphism which eventually produces two different sized proteins. The VDR polymorphism can play a role in the development of ASD by influencing the action pathway of vitamin D [36-40].

Identified genotypes of Fok-1(rs2228570) are FF, Ff, ff; Bsm-I (rs1544410) are BB, Bb, bb; Taq I (rs731236) genotypes are TT, Tt, tt, and Apa-I (rs79752320 are AA, Aa, and aa). Allele t and A were found to be predominant in ASD patients [36-38]. An association between female gender and allele T was found supporting the study that autism is more common in a male gender as T allele is less frequently present in a male child [36, 40-41]. There were few studies which founded an association between VDR gene polymorphisms and serum 25(OH)D level. The responsible alleles for ASD are interestingly associated with increased levels of serum 25(OH)D while some studies showed that lower serum levels of 25(OH)D increase the risk of ASD [39]. Coşkun et al. showed in his study the connection between FokI TT(ff), TaqI CC(tt), BsmI AA(BB) genotypes and ASD [37]. FokI genotype ff is related to high 25(OH)D levels in ASD and presence of one F allele lowers the 1,25(OH)D level which indicates that FokI has an imminent role in vitamin D metabolism and ASD [41-42]. FokI is present in the exon-2 regions of the VDR which is a significant zone to regulate translation. The F allele of FokI polymorphism delivers shorter VDR of 424 amino acids, while the f allele one is longer containing 427 amino acids. The BsmI and Apa I polymorphisms located in intron 8 and TaqI are close to the 3'UTR region of the exon-9. The UTR region controls the VDR mRNA stability and post-transcriptional process which support the role of TaqI polymorphism in alteration of protein structure and vitamin D binding specificity [37, 39, 42].

Paternal homozygous VDR TaqI and BsmI variants showed some relation with ASD [40, 43]. The male reproductive tract has VDR and vitamin D metabolizing enzymes [44-45]. Animal studies exhibited the role of VDR in sperm production and motility [46]. VDR and vitamin D metabolizing enzymes express together in the mature neck of spermatozoa during the late stages of spermatogenesis [45]. Reduced sperm motility is the reason behind aneuploid sperm and sperms with chromosomal abnormalities which can negatively influence developing embryos [44]. One of the studies mentioned that VDR role in sperm production and motility has a more significant effect than a member of the steroid receptor superfamily in influencing DNA translation and transcription [47]. Another study was done by O'Roak et al. in 2012, which demonstrated that de novo mutation had a significant role in the development of ASD and de novo point mutations in protein coding regions are mostly of paternal origin [48].

Autism spectrum disorder is also a disorder of innate immunity [6]. In ASD patients genes related to innate immunity like natural killer (NK) and cytotoxic (CD+8) cells are increased whereas the genes regulating neurodevelopment get reduced. Increased rate of autoimmune disorders in the families of patients with ASD get reported. Anti-fetal brain antibodies are detected in pregnant mothers which can be a potential cause of ASD. The anti-inflammatory action of vitamin D has an active role in regulating the immune function of the human body. The increased risk of getting tuberculosis infection in patients with 1,25(OH)2D3 deficiency supports the role of VDR and 1,25(OH)2D3 in innate immunity [40, 49].

Role of VDR polymorphism in ASD is still poorly understood. However, VDR gene polymorphism is identified in several neuropsychiatric disorders like schizophrenia, bipolar disorder, ADHD, neurodegenerative diseases, and neuroimmune diseases [50]. However. there are a few data available for VDR polymorphism’s relation with ASD.

## Conclusions

Understanding the pathophysiology of ASD remains a big challenge. There are many etiologies like environmental, genetic, nutritional factors which have been reported in several studies done till date. In this literature review, we discussed the association of VDR changes with ASD. Despite selecting all the relevant articles, this review might lack some other related information included in the excluded articles. Evidence compiled from animal and human studies showed that vitamin D plays an essential role in developing fetal brain and any abnormality of vitamin D metabolism in preconception or during pregnancy can affect the fetal neurodevelopmental outcome. VDR polymorphism in certain vitamin D gene variants can influence vitamin D uptake and metabolism in the human body. It needs more research to get a proper conclusion to answer the question on how VDR changes can be a factor in ASD. Very few human studies are available related to VDR polymorphism in ASD. More human studies with larger data sample should be conducted in ASD prone patient population to confirm the findings in this article and to implement clinical recommendations based on pieces of evidence to treat and prevent ASD.
